# Supplementary material for: Paternal Diet before Conception and Its Social Determinants in the Elfe Cohort
Source: Nutrients. 2022 Sep 27;14(19):4008. doi: 10.3390/nu14194008 (PMC9570592; doi:10.3390/nu14194008)
Supplement: Supplementary file 1 [file nutrients-14-04008-s001.zip › nutrients-1898531-supplementary.pdf]

Supplementary Table S1. Descriptive statistics on paternal diet

	Daily frequency
Bread/rusks	1.1 (0.8)
Whole grains cereals	0.3 (0.5)
Milk	0.5 (0.5)
Dairy products	0.8 (0.6)
Dairy deserts	0.3 (0.4)
Cheese	0.8 (0.6)
Cooked vegetables	0.5 (0.4)
Raw vegetables/salad	0.6 (0.6)
Fruit	0.7 (0.7)
Nuts	0.1 (0.1)
Pasta/Rice/Semolina	0.5 (0.3)
Potatoes	0.2 (0.2)
French Fries	0.2 (0.2)
Fish[1]	0.3 (0.2)
Eggs	0.2 (0.2)
Ham/Poultry	0.4 (0.3)
Meat	0.4 (0.3)
Processed meat	0.2 (0.3)
Pizza/Pies/Sandwiches	0.2 (0.2)
Pastries/Biscuits	0.3 (0.3)
Cakes	0.2 (0.3)
Candies/Chocolate	0.3 (0.4)
Chips/Crackers	0.2 (0.2)
Olive oil	0.5 (0.5)
Diet products[4]	0.3 (0.7)
Prepacked foods	0.4 (0.4)
Sweetened beverages[2]	0.9 (0.8)
Beer	0.3 (0.4)
Wine	0.4 (0.6)
Other alcohols[3]	0.3 (0.5)

[1] both caned and fresh, [2] including fruit juices, [3] short drinks, cider and strong alcohol, [4] diet soda, low sugar products and low-fat products

Supplementary Table S2. Unadjusted associations between paternal and household characteristics and paternal diet (n=998): the ELFE study, 2011

	Diverse diet	Balanced	Alcohol	Snacking	Bread and cheese	Processed products
Paternal characteristics						
Age, years						
< 27	0.11 [-0.15 ; 0.36]†	-0.35 [-0.59 ; -0.10]	-0.20 [-0.46 ; 0.05]	-0.14 [-0.40 ; 0.11]	-0.27 [-0.52 ; -0.01]	0.03 [-0.22 ; 0.29]
27-31	0 [Ref]	0 [Ref]	0 [Ref]	0 [Ref]	0 [Ref]	0 [Ref]
32-36	0.05 [-0.10 ; 0.21]	0.24 [0.09 ; 0.39]	-0.10 [-0.26 ; 0.05]	0.06 [-0.10 ; 0.21]	0.03 [-0.13 ; 0.18]	-0.01 [-0.17 ; 0.15]
≥ 37	-0.04 [-0.21 ; 0.13]	0.57 [0.40 ; 0.73]	0.03 [-0.13 ; 0.20]	0.14 [-0.03 ; 0.31]	0.08 [-0.10 ; 0.25]	0.00 [-0.17 ; 0.16]
Education level						
Up to upper secondary school	0.04 [-0.12 ; 0.20]	-0.59 [-0.74 ; -0.43]	0.07 [-0.09 ; 0.23]	-0.15 [-0.31 ; 0.00]	0.17 [0.01 ; 0.33]	0.13 [-0.03 ; 0.28]
Intermediate	0.08 [-0.10 ; 0.26]	-0.36 [-0.54 ; -0.19]	-0.01 [-0.19 ; 0.18]	-0.13 [-0.31 ; 0.05]	0.15 [-0.04 ; 0.33]	0.16 [-0.02 ; 0.34]
3-year university degree	0.16 [-0.05 ; 0.37]	-0.10 [-0.30 ; 0.10]	0.01 [-0.20 ; 0.22]	0.02 [-0.18 ; 0.23]	0.02 [-0.18 ; 0.23]	0.05 [-0.16 ; 0.26]
At least 5-year university degree	0 [Ref]	0 [Ref]	0 [Ref]	0 [Ref]	0 [Ref]	0 [Ref]
Country of birth						
Born abroad	-0.14 [-0.43 ; 0.15]	0.11 [-0.18 ; 0.40]	0.04 [-0.25 ; 0.33]	0.19 [-0.10 ; 0.48]	-0.51 [-0.80 ; -0.22]	-0.23 [-0.52 ; 0.07]
Born in France	0 [Ref]	0 [Ref]	0 [Ref]	0 [Ref]	0 [Ref]	0 [Ref]
Older children in household						
First child	0 [Ref]	0 [Ref]	0 [Ref]	0 [Ref]	0 [Ref]	0 [Ref]
At least one other child	0.13 [0.00 ; 0.25]	0.09 [-0.04 ; 0.21]	-0.14 [-0.26 ; -0.01]	-0.07 [-0.19 ; 0.06]	0.21 [0.09 ; 0.34]	-0.08 [-0.20 ; 0.05]
Employment						
Employed	0 [Ref]	0 [Ref]	0 [Ref]	0 [Ref]	0 [Ref]	0 [Ref]
Unemployed	0.17 [-0.04 ; 0.39]	-0.09 [-0.31 ; 0.12]	0.29 [0.08 ; 0.51]	0.03 [-0.19 ; 0.24]	-0.11 [-0.33 ; 0.10]	-0.12 [-0.33 ; 0.10]
Household characteristics						
Income per consumption unit, per month						
< € 1112	0.05 [-0.18 ; 0.29]	-0.23 [-0.46 ; -0.01]	0.11 [-0.13 ; 0.34]	-0.07 [-0.29 ; 0.16]	-0.21 [-0.43 ; 0.02]	-0.27 [-0.51 ; -0.03]
€1112–1500	0 [Ref]	0 [Ref]	0 [Ref]	0 [Ref]	0 [Ref]	0 [Ref]
€1501–1944	0.07 [-0.10 ; 0.25]	0.09 [-0.08 ; 0.26]	-0.09 [-0.26 ; 0.08]	-0.04 [-0.21 ; 0.14]	-0.12 [-0.29 ; 0.05]	-0.04 [-0.22 ; 0.14]
€1945–2500	-0.13 [-0.32 ; 0.05]	0.29 [0.11 ; 0.48]	-0.10 [-0.28 ; 0.09]	0.06 [-0.13 ; 0.24]	-0.21 [-0.39 ; -0.02]	-0.01 [-0.20 ; 0.17]
> €2500	-0.19 [-0.40 ; 0.02]	0.52 [0.31 ; 0.74]	-0.10 [-0.31 ; 0.11]	0.06 [-0.15 ; 0.27]	-0.12 [-0.33 ; 0.10]	0.00 [-0.21 ; 0.21]
City size						
Rural area	0.22 [0.08 ; 0.36]	-0.17 [-0.31 ; -0.03]	-0.04 [-0.18 ; 0.10]	-0.03 [-0.17 ; 0.11]	0.31 [0.17 ; 0.45]	0.06 [-0.08 ; 0.20]
Urban area	0 [Ref]	0 [Ref]	0 [Ref]	0 [Ref]	0 [Ref]	0 [Ref]

† Values are estimates [95% CI] from simple linear regressions.

Supplementary Table S3. Unadjusted associations between maternal characteristics and paternal diet (n=998): the ELFE study, 2011

	Diverse diet	Balanced	Alcohol	Snacking	Bread and cheese	Processed products
Maternal characteristics						
Age difference with father						
Older mother	0.27 [0.06 ; 0.48] †	0.07 [-0.13 ; 0.28]	-0.05 [-0.26 ; 0.16]	0.02 [-0.19 ; 0.23]	0.00 [-0.20 ; 0.21]	-0.11 [-0.32 ; 0.09]
Same age	0 [Ref]	0 [Ref]	0 [Ref]	0 [Ref]	0 [Ref]	0 [Ref]
Mother 1-2 years younger	0.18 [-0.02 ; 0.39]	0.14 [-0.07 ; 0.35]	-0.08 [-0.29 ; 0.12]	0.02 [-0.19 ; 0.23]	0.10 [-0.11 ; 0.31]	-0.08 [-0.29 ; 0.12]
Mother 3-4 years younger	0.22 [0.01 ; 0.44]	0.07 [-0.15 ; 0.29]	-0.02 [-0.24 ; 0.20]	0.20 [-0.01 ; 0.42]	0.11 [-0.11 ; 0.33]	-0.26 [-0.48 ; -0.04]
Mother at least 5 years younger	0.00 [-0.24 ; 0.24]	0.16 [-0.07 ; 0.39]	0.01 [-0.22 ; 0.24]	0.11 [-0.12 ; 0.35]	0 [-0.25 ; 0.24]	0.00 [-0.23 ; 0.23]
Education level difference with father						
Equivalent level	0 [Ref]	0 [Ref]	0 [Ref]	0 [Ref]	0 [Ref]	0 [Ref]
Father's > Mother's level	-0.06 [-0.22 ; 0.11]	-0.01 [-0.17 ; 0.16]	-0.06 [-0.23 ; 0.10]	0.09 [-0.08 ; 0.25]	0.02 [-0.15 ; 0.20]	-0.08 [-0.25 ; 0.08]
Father's < Mother's level	0.02 [-0.13 ; 0.17]	-0.13 [-0.28 ; 0.01]	-0.03 [-0.17 ; 0.11]	0.13 [-0.01 ; 0.28]	0.13 [-0.02 ; 0.28]	-0.03 [-0.18 ; 0.12]
Country of birth						
Born abroad	-0.09 [-0.37 ; 0.18]	0.25 [-0.02 ; 0.53]	0.10 [-0.17 ; 0.38]	-0.03 [-0.30 ; 0.25]	-0.26 [-0.53 ; 0.02]	-0.15 [-0.43 ; 0.12]
Born in France	0 [Ref]	0 [Ref]	0 [Ref]	0 [Ref]	0 [Ref]	0 [Ref]
Employment during pregnancy						
Employed	0 [Ref]	0 [Ref]	0 [Ref]	0 [Ref]	0 [Ref]	0 [Ref]
Unemployed	0.03 [-0.18 ; 0.24]	-0.14 [-0.34 ; 0.06]	0.16 [-0.05 ; 0.36]	0.23 [0.03 ; 0.43]	-0.06 [-0.26 ; 0.15]	0.04 [-0.16 ; 0.24]
Out of the labor force	-0.12 [-0.34 ; 0.10]	-0.09 [-0.30 ; 0.12]	-0.12 [-0.34 ; 0.09]	-0.04 [-0.26 ; 0.18]	-0.11 [-0.33 ; 0.10]	-0.10 [-0.32 ; 0.12]

† Values are estimates [95% CI] from simple linear regressions.

Supplementary Table S4. Unadjusted associations between paternal health-related characteristics and paternal diet (n=998): the ELFE study, 2011

	Diverse diet	Balanced	Alcohol	Snacking	Bread and cheese	Processed products
Health-related characteristics						
Smoking status						
No smoker	0 [Ref]	0 [Ref]	0 [Ref]	0 [Ref]	0 [Ref]	0 [Ref]
Smoker	-0.06 [-0.22 ; 0.10] †	-0.26 [-0.42 ; -0.10]	0.49 [0.34 ; 0.64]	-0.01 [-0.16 ; 0.13]	0.02 [-0.12 ; 0.17]	0.16 [0.01 ; 0.31]
BMI						
<18.5 kg/m^2	0.07 [-0.68 ; 0.81]	-0.34 [-1.09 ; 0.40]	-0.26 [-1.01 ; 0.49]	0.45 [-0.29 ; 1.19]	0.34 [-0.41 ; 1.08]	-0.36 [-1.10 ; 0.38]
18.5-24.9 kg/m^2	0 [Ref]	0 [Ref]	0 [Ref]	0 [Ref]	0 [Ref]	0 [Ref]
25-29.9 kg/m^2	-0.04 [-0.18 ; 0.09]	-0.02 [-0.15 ; 0.12]	-0.06 [-0.20 ; 0.08]	-0.24 [-0.38 ; -0.11]	-0.04 [-0.18 ; 0.10]	0.21 [0.07 ; 0.34]
At least 30 kg/m^2	-0.08 [-0.31 ; 0.14]	-0.22 [-0.44 ; 0.01]	0.02 [-0.20 ; 0.25]	-0.21 [-0.43 ; 0.02]	-0.21 [-0.44 ; 0.01]	0.22 [0.00 ; 0.45]
Regular physical activity[1]						
No	0 [Ref]	0 [Ref]	0 [Ref]	0 [Ref]	0 [Ref]	0 [Ref]
Yes	0.11 [-0.02 ; 0.24]	-0.02 [-0.15 ; 0.11]	0.05 [-0.08 ; 0.18]	0.03 [-0.10 ; 0.17]	0.03 [-0.10 ; 0.15]	-0.16 [-0.30 ; -0.03]
Regular walking[2]						
No	0 [Ref]	0 [Ref]	0 [Ref]	0 [Ref]	0 [Ref]	0 [Ref]
Yes	0.06 [-0.06 ; 0.19]	0.22 [0.09 ; 0.34]	-0.03 [-0.16 ; 0.10]	0.05 [-0.08 ; 0.18]	-0.04 [-0.17 ; 0.09]	-0.01 [-0.13 ; 0.12]
Restrictive diet						
No	0 [Ref]	0 [Ref]	0 [Ref]	0 [Ref]	0 [Ref]	0 [Ref]
Yes	-0.05 [-0.32 ; 0.23]	0.46 [0.18 ; 0.73]	-0.29 [-0.57 ; -0.02]	-0.59 [-0.86 ; -0.32]	-0.33 [-0.61 ; -0.06]	1.03 [0.76 ; 1.29]

† Values are estimates [95% CI] from simple linear regressions.

[1] at least 1 hr/week, [2] at least 30 min/day, 5 days/week

Supplementary Table S5. Unadjusted associations between maternal diet and paternal diet (n=998): the ELFE study, 2011

	Diverse diet	Balanced	Alcohol	Snacking	Bread and cheese	Processed products
Maternal diet during pregnancy						
Energy intake (per 100 kcal)	0.03 [0.02 ; 0.03] †	-0.01 [-0.02 ; -0.01]	0.00 [-0.01 ; 0.01]	0.00 [-0.01 ; 0.01]	0.00 [-0.01 ; 0.01]	0.00 [-0.01 ; 0.01]
PCA pattern 1: Western diet	0.25 [0.18 ; 0.32]	-0.26 [-0.33 ; -0.19]	-0.02 [-0.09 ; 0.06]	-0.06 [-0.13 ; 0.01]	-0.10 [-0.17 ; -0.04]	-0.03 [-0.10 ; 0.04]
PCA pattern 2: Healthy diet	0.17 [0.09 ; 0.24]	0.39 [0.32 ; 0.46]	0.01 [-0.06 ; 0.08]	0.03 [-0.04 ; 0.10]	0.00 [-0.07 ; 0.07]	-0.04 [-0.11 ; 0.03]
PCA pattern 3: Bread and toppings	0.10 [0.03 ; 0.17]	0.13 [0.06 ; 0.20]	0.01 [-0.07 ; 0.08]	0.12 [0.04 ; 0.20]	0.15 [0.08 ; 0.21]	0.05 [-0.02 ; 0.13]
PCA pattern 4: Processed products	0.01 [-0.07 ; 0.09]	-0.04 [-0.13 ; 0.05]	0.04 [-0.04 ; 0.12]	0.03 [-0.04 ; 0.11]	-0.04 [-0.12 ; 0.03]	0.16 [0.09 ; 0.23]
PCA pattern 5: Milk and breakfast cereals	-0.04 [-0.11 ; 0.04]	-0.05 [-0.13 ; 0.02]	-0.12 [-0.19 ; -0.04]	-0.04 [-0.11 ; 0.03]	-0.08 [-0.15 ; -0.01]	0.16 [0.09 ; 0.23]
Alcohol in early pregnancy						
Never during this period	0 [Ref]	0 [Ref]	0 [Ref]	0 [Ref]	0 [Ref]	0 [Ref]
<1 glass/week	0.08 [-0.08 ; 0.23]	0.17 [0.02 ; 0.33]	0.15 [-0.02 ; 0.31]	0.06 [-0.10 ; 0.21]	-0.07 [-0.23 ; 0.08]	0.10 [-0.07 ; 0.26]
At least 1 glass/week	0.12 [-0.03 ; 0.28]	0.38 [0.22 ; 0.54]	0.49 [0.33 ; 0.65]	0.21 [0.05 ; 0.37]	0.14 [-0.02 ; 0.30]	0.14 [-0.02 ; 0.30]

† Values are estimates [95% CI] from simple linear regressions.
